# Turkish validity and reliability study of dialysis-related stigma scale

**DOI:** 10.3389/fpsyg.2026.1796321

**Published:** 2026-07-29

**Authors:** Esra Başer Akın, Safiye Yanmış Karaca, Mukadder Mollaoğlu

**Affiliations:** 1 Department of Nursing, Faculty of Health Science, Sivas Cumhuriyet University, Sivas, Türkiye; 2 Department of Nursing, Faculty of Health Science, Erzincan Binali Yıldırım University, Erzincan, Türkiye

**Keywords:** dialysis, nursing, reliability, stigma, validity

## Abstract

**Introduction:**

Patients’ psychological well-being, social functioning, and treatment adherence are all adversely impacted by the stigma associated with dialysis. For the purpose of recognizing stigma and directing nursing interventions, accurate and culturally relevant measurement instruments are crucial. The purpose of this study was to assess the validity and reliability of the Dialysis-Related Stigma Scale (DRSS) in Turkish.

**Methods:**

This methodological study was conducted at a private dialysis facility in Sivas, Turkiye, between July and September 2025. Data were collected from 92 participants using the “Patient Information Form” and the “DRSS.” Language validity, content validity, item analyses, internal consistency tests, and test–retest reliability analyses were carried out using SPSS 23.0 and AMOS 23.0 software during the scale’s validity and reliability stage. Content validity was evaluated using the Content Validity Index (CVI), construct validity was assessed using exploratory and confirmatory factor analyses, and reliability was examined using internal consistency and test–retest analyse analyses.

**Results:**

With a Cronbach’s alpha coefficient of 0.89 (McDonald’s omega = 0.894), the scale demonstrated excellent internal consistency. The scale’s content validity index of the Turkish version was 0.91. Test–retest analysis revealed a strong positive correlation (*r* = 0.967, *p* < 0.05).

**Discussion:**

Finally, the Turkish version of the DRSS was found to be a valid and reliable instrument for measuring stigma in dialysis patients.

## 1 Introduction

End-stage renal disease constitutes a significant public health issue with rapidly increasing global prevalence (Rafferty et al., 2025). Among available renal replacement therapies, hemodialysis remains the most frequently utilized modality worldwide and in Turkiye (United States Renal Data System., 2024).

While hemodialysis aims to replace essential kidney functions, patients often experience a considerable burden of treatment-related complications (Habas et al., 2021). Common clinical manifestations include hemodialysis-associated infections, cardiovascular events, respiratory and gastrointestinal complications, and neuromuscular symptoms (Hiyamuta et al., 2021). Anemia, metabolic bone disease, pruritus, sleep disorders, and pain are also common (Habas et al., 2021). In addition to these physical complications, patients also experience negative psychological experiences such as anxiety, depression, and stigma, which seriously disrupt their quality of life (Li et al., 2024). While stigma has been widely recognized in the context of infectious diseases, increasing evidence suggests that it is also a significant psychosocial concern among individuals with chronic non-communicable diseases, including chronic kidney disease (Rai et al., 2020).

Stigma refers to negative attitudes, prejudice, and discrimination toward individuals or groups perceived as socially discrediting (Yeni, 2023). In the context of chronic illness, stigma may emerge when disease-related characteristics, treatment requirements, or functional limitations distinguish individuals from perceived social norms, resulting in adverse psychological, social, and health-related outcomes (Akbari et al., 2023; Yeni, 2023). The progressive nature of chronic diseases, the need for lifelong treatment, changes in physical appearance, and limitations in daily activities and social roles may increase the risk of experiencing stigma (Best and Arseniev-Koehler, 2023). Individuals who experience stigma often report feelings of shame, guilt, distress, and social isolation, and they may internalize negative societal attitudes, perceiving themselves as devalued or marginalized within society (Woo et al., 2021; Rai et al., 2020). Furthermore, stigma has been associated with decreased healthcare utilization, poor treatment adherence, impaired self-management, and an increased risk of psychological problems among individuals living with chronic diseases (Salih et al., 2022; Perugino et al., 2022).

Patients receiving renal replacement therapy, particularly hemodialysis, have significantly lower levels of physical, mental, social, and economic well-being compared with patients who have undergone kidney transplantation and with the general population (Sułkowski et al., 2024). Comorbidities, depression, and severe symptoms frequently contribute to a decline in well-being and quality of life among patients undergoing dialysis treatment (Lu et al., 2024). Furthermore, hopelessness, anxiety, depression, and inadequate coping strategies lead to passivity and self-restriction in the daily lives of dialysis patients (Montalescot et al., 2021). Dialysis patients often report low self-esteem, perceive themselves as a burden to their families, and feel that they do not contribute to family life. As a result, they tend to avoid making plans for the future and limit social interactions (Rikos et al., 2023; Safi et al., 2024).

Several studies have demonstrated that patients undergoing hemodialysis experience moderate-to-substantial levels of stigma related to the long-term and restrictive nature of treatment and its psychosocial consequences (Zhang et al., 2022; Li et al., 2023). Zhang et al. (2022) assessed stigma among 97 young and middle-aged maintenance hemodialysis patients in a cross-sectional study and reported moderate levels of stigma, particularly among patients with longer dialysis duration, lower income levels, and unemployment. Another cross-sectional study conducted in China examined stigma and its associated factors among 204 dialysis patients. Stigma was assessed using the Social Impact Scale, and the findings indicated that patients receiving kidney dialysis experienced moderate levels of stigma. Stigma was negatively associated with hope and social support and positively associated with perceived stress and fear of disease progression (Li et al., 2023). In a more recent cross-sectional study, Song et al. (2026) investigated heterogeneity in stigma experiences among 558 maintenance hemodialysis patients using latent profile analysis and symptom network analysis. Stigma was measured using the Social Impact Scale. The authors identified three distinct stigma subgroups (low, moderate, and high) and demonstrated that higher stigma levels were associated with greater symptom burden and more complex symptom network patterns.

Dialysis-related stigma has been associated with poorer quality of life, depressive symptoms, social isolation, and lower treatment adherence. Furthermore, concerns about being perceived as different, burdensome, or socially disadvantaged may negatively affect patients’ psychological well-being and social functioning (Li et al., 2024; Zorba et al., 2024).

Increasing evidence indicates that stigma is prevalent across various chronic non-communicable diseases, including diabetes, chronic kidney disease, chronic obstructive pulmonary disease, neurological disorders, and mental disorders. This adversely affects psychological well-being, social participation, healthcare utilization, and quality of life (Akyirem et al., 2024; Salih et al., 2022; Woo et al., 2021; Rai et al., 2020). As the psychosocial burden of dialysis-related stigma has become increasingly recognized, assessing stigma among individuals undergoing dialysis treatment has gained importance (Zhuo et al., 2025; Li et al., 2024). Following a comprehensive search in PubMed, Scopus, Web of Science, and Google Scholar using relevant keywords related to dialysis stigma and scale adaptation, no Turkish-language tool validated for measuring dialysis-related stigma in patients receiving dialysis treatment was identified. In the international context, Sugisawa et al. (2023) developed the DRSS to measure stigma among dialysis patients in Japan. The original scale showed excellent internal consistency (Cronbach’s α = 0.86) and comprised 9 items categorized into three dimensions: “blame and judgment,” “treated differently,” and “self-stigma.” The DRSS exhibited satisfactory psychometric properties in its original validation study, offering a disease-specific method for evaluating dialysis-related stigma (Sugisawa et al., 2023). Consequently, this study aims to adapt the DRSS into Turkish and assess its validity and reliability among patients receiving dialysis treatment in Turkiye.

## 2 Materials and methods

### 2.1 Participants

This methodological validity and reliability study was conducted among patients undergoing treatment between July and September 2025 at the private ONR dialysis center in Sivas, Turkiye. The private ONR Dialysis Center had a capacity of 48 beds and provided services 6 days a week (except for Sundays). No other dialysis unit of similar size served hemodialysis patients in Sivas province. The Private ONR Dialysis Center was chosen because it had the highest hemodialysis patient population in Sivas province. The sample size was determined according to the recommendation that the number of participants should be at least five to ten times the number of items in the scale for factor analysis (Memon et al., 2020). Since the scale consisted of nine items, a minimum sample size of 45–90 participants was required.

The following criteria were employed for inclusion.

- Being over 18 years of age
- Receiving hemodialysis treatment for at least 3 months
- Having no severe hearing or visual impairment that would prevent questionnaire completion
- Agreeing to participate in the study

**FIGURE 1:**
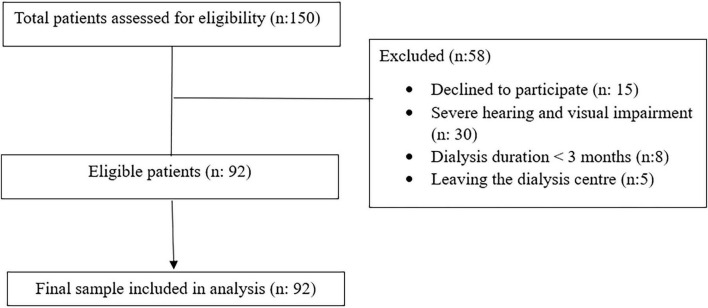
Flow diagram.

Taking into account the item-to-participant ratio, the sample size was determined to be 92 patients who met the inclusion criteria. During the study period, 15 patients declined to participate. Of the remaining patients, 30 were excluded due to severe hearing and visual impairments that wouldn’t reply questionnaire, 8 were excluded because they had undergone dialysis treatment for less than 3 months, and 5 left the dialysis center. Consequently, the study was completed with 92 participants. Figure 1 shows the flow diagram of patient selection through the research process.

### 2.2 Measures

Data were collected by researchers using the “*Patient Information Form*” and the “*DRSS*” through face-to-face interviews.

#### 2.2.1 Patient information form

The form consisted of 15 items designed to collect participants’ sociodemographic and disease-related characteristics. Sociodemographic variables included age, gender, marital status, education level, employment status, occupation, income level, living conditions, participation in social activities, and dependency status in activities of daily living. Disease-related variables included the presence of other chronic diseases, number of weekly dialysis sessions, duration of chronic kidney disease, duration of dialysis treatment, and type of vascular access.

#### 2.2.2 Dialysis-related stigma scale (DRSS)

The scale was developed based on an existing Type 2 Diabetes Stigma Assessment Scale consisting of three factors: “blame and judgment,” “treated differently,” and “self-stigma” (Browne et al., 2016). The scale consisted of nine items, and all items were scored on a 5-point Likert scale ranging from “0” (I strongly disagree) to “4” (I strongly agree). Total scores were calculated by summing the individual scores of all items. Items 1, 2, and 3 measure treated differently in care, items 4, 5, and 6 measure blame and judgement, and items 7, 8, and 9 measure self-stigma factors. The total scale score was obtained by summing all items. As the total score increases, the level of stigma increases. The Cronbach’s alpha value of the scale was found to be 0.86 (Sugisawa et al., 2023). In this study, the Cronbach’s alpha coefficient was found to be 0.89 (McDonald’s omega = 0.894).

### 2.3 Implementation of data collection forms

The data were collected via a single face-to-face interview based on self-reporting by the participants. The questionnaire was re-administered 2 weeks later to 30 participants who could be reached for the second assessment from the entire sample. Patients were asked to choose a code name for themselves and to write this code on the form during the test-retest procedure, without writing their names on the forms. Participants were informed about the purpose of the study and assured of the confidentiality and anonymity of their responses to minimize response bias. The scale was administered in a standardized manner. All items were read to the participants verbatim without any modification, explanation, or interpretation that could influence their responses. The researcher followed a uniform instruction protocol for all participants.

### 2.4 Scale validity and reliability stages

Permission was obtained from the developer of the DRSS for the Turkish adaptation of the scale. DRSS was originally developed in English by Japanese researchers (Sugisawa et al., 2023), and the Turkish adaptation was based on the English version of the scale reported in the original publication. For the language adaptation of the scale, the translation-back translation technique and equivalence testing were performed. Two academics with knowledge of English and an English linguist translated it from English into Turkish. This form was then retranslated back into English using the back-translation method. The translations were reviewed by researchers, and the original English scale was compared with the scale subsequently translated into English. To ensure semantic and conceptual equivalence, wording, sentence structure, and cultural appropriateness were reviewed during the adaptation process. Since the majority of the items were linguistically and culturally appropriate for the Turkish context, substantial modifications were not required. The scale items were translated into Turkish while maintaining their original meaning and conceptual accuracy. Minor wording adjustments were made where necessary to improve clarity and comprehensibility for Turkish patients.

Content validity was assessed by a panel of five nursing faculty members with expertise in Medical Nursing and Psychiatric Nursing, substantial clinical experience in hemodialysis care, and expertise in scale validity and reliability assessment. Each expert on the panel has at least 10 years of professional experience in their respective field. The Davis technique was employed to evaluate the content validity of expert opinions. In this technique, expert evaluations are rated on a four-point scale: 1 (inappropriate), 2 (appropriate), 3 (very appropriate), and 4 (completely appropriate). According to the literature data, a CVI of ≥ 0.80 indicates adequate content validity (Davis, 1992).

To assess the suitability of the sample for factor analysis, the Kaiser-Meyer-Olkin (KMO) measure and Bartlett’s Test of Sphericity were applied (Shrestha, 2021). Exploratory Factor Analysis (EFA) was conducted to examine the underlying factor structure of the measurement tool. Subsequently, Confirmatory Factor Analysis (CFA) was performed to verify the identified factor structure and evaluate its construct validity (Orcan, 2018; Yaşlıoğlu, 2017).

After confirming the suitability of the data for factor analysis, principal components analysis with Varimax orthogonal rotation was used to determine the factor structure of the scale. Based on the literature, factor loadings of ≥ 0.30 were considered acceptable, and items with loadings below this threshold were excluded from the scale (Hair et al., 2016).

Reliability was assessed using internal consistency analysis (Cronbach’s alpha), the split-half method, and item analysis. A Cronbach’s alpha coefficient of ≥ 0.70 was considered indicative of adequate internal consistency (DeVellis et al., 2021). Item–total correlation coefficients were calculated to examine the relationship between individual item scores and the total scale score. In line with the literature, an item–total correlation coefficient of ≥ 0.30 was accepted as the criterion for item retention.

### 2.5 Statistical analysis

The data obtained in the study were analyzed using IBM Statistical Package for the Social Sciences (SPSS) 23 (licensed program) and Analysis of Moment Structures (AMOS) 23 programs. Before analysis, the dataset was examined for missing data and normality. No missing data was detected. Normality of the data was assessed using Skewness and Kurtosis values. Descriptive statistics were calculated using percentages, mean, and standard deviation. In the validity study of the DRSS, content validity was evaluated based on expert opinions using the CVI according to the Davis technique. Construct validity was assessed using EFA and CFA, supported by the KMO test and Bartlett’s Test of Sphericity. Reliability was examined using Cronbach’s alpha coefficient, McDonald’s omega, the split-half method, and item-total correlation analysis.

### 2.6 Research ethics and patient consent

This study was conducted in accordance with the principles of the Declaration of Helsinki, and ethical approval was obtained from the Clinical Research Ethics Committee of Sivas Cumhuriyet University (approval number: 2025-07/64, dated 10 July 2025). Written institutional permission was also obtained from the institution where the study was conducted. Permission was obtained via email from the author who developed the DRSS to conduct the Turkish validity-reliability analysis. The purpose of the study was explained to the patients, and they were informed that the data obtained from the study would be stored securely. Written consent was obtained from the patients before the collection of research data.

## 3 Results

The mean age of the participants was 62.14 ± 13.11 years. Of the participants, 51.1% were female, 64.1% were married, 52.2% had completed primary education, 47.8% were housewives, and 51.1% reported a moderate income level. More than half of the participants (55.4%) had been diagnosed with chronic kidney disease for 1–5 years, while 57.6% had been receiving hemodialysis treatment for 1–5 years. The most frequently used vascular access route was an arteriovenous fistula (88.0%). Regarding social participation and functional status, 32.6% of the participants reported occasional participation in social activities, 57.6% were independent in activities of daily living, and 76.1% lived with their spouse or family members (Table 1).

**TABLE 1:** Participants’ sociodemographic characteristics.

Variables	Mean ± SD	*n*	%
Age	62.14 ± 13.11		
Gender			
Female		47	51.1
Male		45	48.9
Marital status			
Married		59	64.1
Unmarried		33	35.9
Educational level			
Illiterate		15	16.3
Literate		8	8.7
Primary school		48	52.2
High school		18	19.6
University		3	3.3
Working status			
House wife		44	47.8
Student		1	1.1
Retired		39	42.4
Other		8	8.7
Income status			
Sufficient		30	32.6
Moderate		47	51.1
Insufficient		15	16.3
CKD vintage			
1–5 years		51	55.4
6–10 years		21	22.8
11–15 years		14	15.2
15 years and above		6	6.5
Dialysis vintage			
1–5 years		53	57.6
6–10 years		22	23.9
11–15 years		11	12
15 years and above		6	6.5
Vascular access			
AV Fistula		81	88.0
Permanent Catheter		5	5.4
Temporary Catheter		6	6.5
Participation status in events			
Never		19	20.7
Rarely		28	30.4
Sometimes		30	32.6
Frequently		15	16.3
Dependency in performing ADL*			
Dependent		6	6.5
Semi-dependent		33	35.9
Independent		53	57.6
Living arrangements			
Alone		11	12.0
With family		70	76.1
With children		11	12.0

*ADL, The Activities of Daily Living.

### 3.1 Validity analysis

The scale’s CVI was determined to be 0.91 based on the assessments of five experts. The scale items were deemed to have sufficient content validity because the CVI was higher than the suggested threshold of 0.80. As a result, nothing was taken off the scale. The KMO measure of sampling adequacy was 0.806, and Bartlett’s Test of Sphericity yielded a χ^2^ value of 995.628, which was statistically significant (*p* < 0.001). These findings indicated that the sample size was adequate and that the data were suitable for factor analysis (Table 2).

**TABLE 2:** Kaiser-Meyer-Olkin and Bartlett test results.

Test		Results	
Kaiser-Meyer-Olkin measure of sampling adequacy		0.806	*p* < 0.001
Bartlett’s Test	Approx. Chi-square	995.628	
	df	36	
	Sig.	< 0.001	

The Varimax Rotation Method was used in the EFA. Although it is acknowledged that modeling correlations among factors may provide more realistic results, particularly in psychosocial constructs, Varimax rotation was preferred at this stage to achieve a clearer and more interpretable factor structure. The analysis revealed a three-factor structure for the scale. Factor 1 (“Treated Differently”), which explained 34.391% of the variance, was loaded by items 1, 2, and 3. Items 7, 8, and 9 loaded on Factor 3 (“Self-Stigma”), accounting for 20.413% of the variance, while items 4, 5, and 6 loaded on Factor 2 (“Blame and Judgment”), accounting for 28.234% of the variance. Collectively, the three-factor structure explained 83.038% of the total variance (Table 3).

**TABLE 3:** Mean score, item-total score correlation coefficients, factor loadings, alpha coefficients, and explained variance of the diagnostic and statistical inventory (DSI).

Item	Factor 1	Factor 2	Factor 3	Item-total correlation
Q1	0.911			0.827*
Q2	0.914			0.841*
Q3	0.910			0.829*
Q4		0.912		0.649*
Q5		0.879		0.708*
Q6		0.744		0.772*
Q7			0.550	0.502*
Q8			0.862	0.429*
Q9			0.776	0.447*
Eigenvalue explained	5.173	1.291	1.010	
Variance (%) total	34.391	28.234	20.413	
Variance (%)	83.038			
Cronbach’s α	0.994	0.913	0.677	Total = 0.897
McDonald’s Omega	0.994	0.914	0.697	Total = 0.894

*p < 0.001, Factor 1: Treated Differently, Factor 2: Blame and Judgment, Factor 3: Self-stigma.

Although modeling correlations among factors may yield more realistic results, particularly in psychosocial constructs, Varimax Rotation was used to achieve a clearer and more interpretable factor structure. The analysis indicated a three-factor structure for the scale.

**FIGURE 2:**
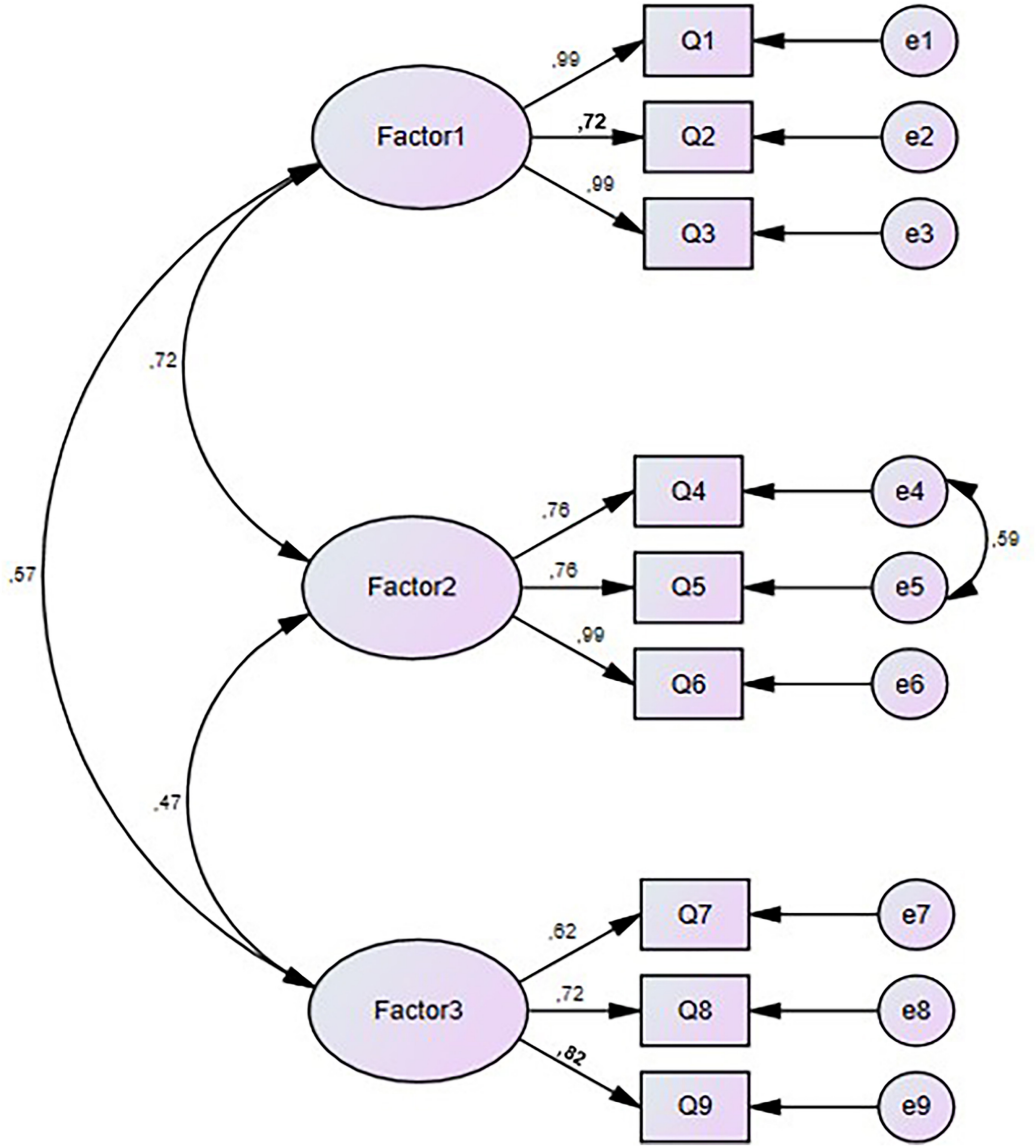
CFA path diagram of the DRSS.

The CFA revealed that the DRSS demonstrated an overall acceptable model fit, with fit indices of χ^2^ = 33.014, df = 23 (*p* < 0.001), χ^2^/df = 1.435, RMSEA = 0.069, CFI = 0.98, TLI = 0.97, IFI = 0.97, GFI = 0.93, AGFI = 0.86, NFI = 0.95, RFI = 0.93, RMR = 0.049, and SRMR = 0.055 (Table 4). The CFA path diagram of the DRSS is shown in Figure 2.

**TABLE 4:** Results of the confirmatory factor analysis.

Fit criteria	Found	Appropriate	Acceptable
*x*^2^/df	1.435	< 2	< 5
RMSEA	0.069	< 0.05	< 0.08
CFI	0.98	> 0.95	> 0.90
TLI	0.97	> 0.95	> 0.90
IFI GFI AGFI	0.98 0.93 0.86	> 0.95 > 0.95 > 0.95	> 0.90 > 0.90 > 0.90
NFI	0.95	> 0.95	> 0.90
RFI	0.93	> 0.95	> 0.90
RMR	0.049	< 0.05	< 0.08
SRMR	0.055	< 0.05	< 0.08

RMSEA, Root Mean Square Error of Approximation; CFI, Comparative Fit Index; TLI, Tucker Lewis Index; IFI, Incremental Fit Index; GFI, Goodness of Fit Index; AGFI, Adjusted Goodness of Fit Index; NFI, Normed Fit Index; RFI, Relative Fit Index; RMR, Root Mean Square Residual; SRMR, Standardized Root Mean Square Residual.

The results of the EFA and CFA consistently supported the original three-factor structure of the DRSS, with no modifications required to achieve an acceptable model fit. These findings provide strong evidence for the construct validity of the Turkish version of the scale and support its cross-cultural equivalence with the original instrument among Turkish patients receiving hemodialysis treatment.

### 3.2 Reliability analysis

In the analyses conducted to determine the reliability of the scale, data were administered again to 30 individuals after 2 weeks, and the pre-test-post-test correlation coefficient was found to be 0.967 for the entire scale; 0.581 for the sub-dimension “Factor 1 (treated differently),” 0.620 for “Factor 2 (blame and judgement),” and 0.866 for “Factor 3 (self-stigma)” (Table 5). These values indicate that the scale has high external reliability and a stable structure.

**TABLE 5:** Test-retest reliability results.

Scale	Measurement	Mean ± SD	*t*	*p*	*r*	*p*
Factor 1	Pretest	4.40 ± 1.42	−2.911	0.700	0.581	< 0.001*
	Posttest	5.10 ± 1.44				
Factor 2	Pretest	5.16 ± 2.56	2.017	0.530	0.620	< 0.001*
	Posttest	4.36 ± 2.41				
Factor 3	Pretest	4.93 ± 2.43	−0.138	0.891	0.866	< 0.001*
	Posttest	4.96 ± 2.63				
Total	Pretest	14.33 ± 5.33	−1.618	0.117	0.967	< 0.001*
	Posttest	14.73 ± 5.16				

t: Dependent Groups t-test, r: Pearson Correlation Coefficient, *p < 0.001.

In addition, internal reliability of the scale was assessed using Cronbach’s alpha coefficient. The Cronbach’s alpha value was 0.897 for the total scale, 0.994 for Factor 1 (treated differently), 0.913 for Factor 2 (blame and judgment), and 0.677 for Factor 3 (self-stigma) (Table 3). These findings indicate that the overall scale possesses strong internal consistency, with satisfactory reliability observed for most subscales (DeVellis et al., 2021; Çokluk et al., 2016). Furthermore, item–total correlation coefficients ranged from 0.55 to 0.91, exceeding the recommended threshold of 0.30 for all items. This finding provides additional evidence for the internal consistency and reliability of the scale.

The reliability of the measurement model was further examined using McDonald’s omega coefficient. The results indicated strong internal consistency across the scale and its sub-dimensions. Specifically, McDonald’s omega (ω) values ranged from 0.697 to 0.994, with an overall omega coefficient of 0.894 for the entire scale. These findings demonstrate that the scale exhibits an acceptable to high level of internal consistency reliability. In particular, the overall omega value (ω = 0.894) indicates strong reliability of the measurement instrument. The relatively high values observed in some sub-dimensions further suggest a high degree of consistency among the items measuring the same construct. Overall, the results support the reliability of the scale and confirm its suitability for further structural analyses.

### 3.3 Measurement model evaluation

The results of the measurement model’s validity and reliability obtained from CFA are presented in Table 6. The findings indicated that standardized factor loadings ranged from 0.62 to 0.99. These values demonstrated that the observed variables adequately reflected their respective latent constructs. Regarding convergent validity, the Average Variance Extracted (AVE) values ranged from 0.525 to 0.826. Since all AVE values exceeded the recommended threshold of 0.50, the measurement model demonstrated acceptable levels of convergent validity (Hair et al., 2016). Composite Reliability (CR) values ranged from 0.766 to 0.933. These results indicated that all constructs exceeded the 0.70 threshold, confirming a high level of internal consistency reliability. Overall, the findings provided evidence that the measurement model was both valid and reliable and that the three-factor structure was empirically supported.

**TABLE 6:** Construct validity and reliability results.

Factors	Item	λ	CR	AVE
Factor 1	1	0.99	0.933	0.826
	2	0.72		
	3	0.99		
Factor 2	4	0.76	0.879	0.712
	5	0.76		
	6	0.99		
Factor 3	7	0.62	0.766	0.525
	8	0.72		
	9	0.82		

CR, Composite Reliability; AVE, Average Variance Extracted; λ, Standardized Loading.

## 4 Discussion

Stigma in chronic illnesses is increasingly recognized as a psychosocial issue, with its perceived, anticipated, and internalized dimensions being the subject of growing research (Akyirem et al., 2024; Browne et al., 2016). Hemodialysis patients constitute a group that is particularly vulnerable to stigma due to the visible and life-altering nature of their treatment. However, the number of studies in this field remains limited (Sugisawa et al., 2023). This study is one of the few adaptation studies addressing dialysis-related stigma specifically within the Turkish context, and the findings are discussed below within the relevant literature.

The findings of this study demonstrated that the DRSS is a valid and reliable instrument for measuring stigma among individuals receiving hemodialysis treatment in Turkish society. Content validity was evaluated using the Davis technique based on the assessments of five field experts, and the CVI was calculated as 0.91. According to the literature, a CVI value greater than 0.80 indicates that the items adequately represent the construct being measured (Davis, 1992). This finding indicated that the scale items are culturally appropriate and sufficiently comprehensive for use in the Turkish context.

According to the results of the EFA conducted as part of construct validity, the scale was found to have a three-factor structure. The three-factor structure identified in this study is theoretically consistent with the multidimensional nature of stigma. Stigma is often conceptualized as encompassing perceived discrimination, internalized stigma, and social judgment (Holmes-Truscott et al., 2018). In this context, the factors identified as ‘treated differently’, ‘blame and judgement’, and “self-stigma” reflect distinct yet interrelated dimensions of the stigma experience among individuals receiving hemodialysis. The scale explained 83.03% of the total variance, which is well above the generally accepted range of 40–60% in the social sciences, indicating a strong factor structure (Hair et al., 2016). A similar multidimensional structure was reported in the original scale development study, suggesting that the Turkish version maintained the conceptual integrity of the original instrument (Sugisawa et al., 2023). However, this relatively high explained variance should be interpreted with caution. While it reflects the structural consistency of the scale, it may also indicate a degree of conceptual proximity among items. In the present study, the multidimensional factor structure, the distribution of item–total correlations, and the variance in reliability coefficients suggest that the scale captures an integrated yet multidimensional experience of stigmatization, rather than representing a narrowly defined or overly redundant construct. Future research with larger and more heterogeneous samples is recommended to further examine the dimensional structure and to ensure that the scale adequately captures the full scope of the construct. CFA conducted following EFA revealed that the three-factor model demonstrated acceptable fit indices and was consistent with the data, thereby confirming the theoretical structure and supporting the construct validity of the scale (Orcan, 2018; Yaşlıoğlu, 2017 Kocatürk-Kapucu et al., 2026). The CFA results indicated an overall adequate to good model fit, with χ^2^ = 33.014, df = 23 (*p* < 0.001), yielding a χ^2^/df ratio of 1.435, which falls within the acceptable range. The RMSEA value of 0.069 indicated an acceptable level of approximation error, while incremental fit indices showed strong model performance (CFI = 0.98, IFI = 0.97, TLI = 0.97, NFI = 0.95, RFI = 0.93). In addition, the RMR (0.049) and SRMR (0.055) values were below the recommended thresholds, further supporting model adequacy. Although the GFI (0.93) and AGFI (0.86) values were slightly below the ideal cutoff of 0.90, they remained within acceptable limits. Overall, these findings indicate that the proposed factor structure of the DRSS is supported by the data and demonstrates acceptable construct validity in accordance with criteria suggested in the literature (Orcan, 2018; Yaşlıoğlu, 2017). However, the relatively small sample size (*n* = 92) should be taken into consideration when interpreting these results. In addition, smaller sample sizes in CFA may increase the risk of overestimating model fit and factor loadings. Therefore, replication of the findings in larger samples is essential to confirm the robustness of the proposed factor structure.

The reliability of the scale was evaluated using both internal consistency and test–retest methods. The overall Cronbach’s alpha coefficient of the scale was 0.897, indicating a high level of internal consistency (DeVellis et al., 2021). Cronbach’s alpha coefficients for the subscales ranged from 0.677 to 0.994. The exceptionally high alpha coefficient observed for the Treated Differently subscale (α = 0.994) indicates a very strong relationship among the items and suggests a high degree of content homogeneity. In scale adaptation and development studies, such findings may be interpreted as evidence supporting the homogeneity of the measured construct (Çokluk et al., 2016). However, reliability coefficients approaching 1.00 may also indicate substantial overlap among items and potential item redundancy (Tavakol and Dennick, 2011). Therefore, although this finding supports the internal consistency of the subscale, it should be interpreted with caution, as excessively high internal consistency may reduce the conceptual breadth of the construct being measured. Future studies are recommended to re-examine the item structure of this subscale to ensure adequate conceptual diversity while maintaining reliability. The variance in reliability coefficients across subscales may be attributable to differences in their conceptual scope and item content. While the exceptionally high reliability coefficient of the Treated Differently subscale may reflect a highly homogeneous construct, relatively lower coefficients observed in other subscales may be associated with the multidimensional and heterogeneous nature of self-stigma experiences across individuals.

The test–retest reliability coefficient was also found to be high, providing evidence for the temporal stability of the scale. Nevertheless, this analysis was conducted using a relatively small subsample (*n* = 30). Although this sample size is consistent with minimum recommendations reported in methodological literature (Kennedy, 2022), the findings should be interpreted cautiously. Future studies employing larger samples are warranted to provide additional evidence regarding the stability of the scale over time.

The Cronbach’s alpha (α = 0.994) and McDonald’s omega (ω = 0.994) coefficients obtained for Factor 1 were exceptionally high. While these findings support the strong internal consistency of the factor, such high reliability estimates in a three-item subscale may also indicate substantial item similarity and potential content overlap. Therefore, although these results provide evidence of reliability, future studies should further examine the discriminative properties of these items and evaluate the possibility of item redundancy.

The overall McDonald’s omega value (ω = 0.894) indicates strong reliability of the measurement instrument. Compared to Cronbach’s alpha, McDonald’s omega is considered a more robust estimator of internal consistency, as it does not assume tau-equivalence among items and provides a more accurate reliability estimate (McNeish, 2018). Although the Cronbach’s alpha value in the present study was very high for Factor 1 (α = 0.994), such values may indicate either excellent internal consistency or potential item redundancy, depending on the construct’s conceptual breadth (Tavakol and Dennick, 2011). However, when interpreted together with McDonald’s omega, the results suggest that the scale demonstrates strong reliability rather than measurement bias. Overall, the reliability analyses confirm that the measurement instrument is suitable for further structural analysis.

The test–retest analysis demonstrated a strong correlation for the overall scale score (*r* = 0.967), indicating excellent temporal stability and consistency of measurements over time. The exceptionally high test–retest coefficient obtained for the total scale further indicates that perceptions of dialysis-related stigma, as measured by the DRSS, are assessed consistently across repeated administrations. The correlation coefficients for the subscales ranged from 0.581 to 0.620, reflecting moderate levels of temporal stability. Although the stability coefficients of the subscales were lower than that of the overall scale, they remained within an acceptable range for repeated measurements. These findings suggest that the Turkish version of the DRSS produces stable scores over time, particularly at the overall scale level. Therefore, the results provide additional evidence supporting the reliability of the instrument for assessing dialysis-related stigma in both clinical and research settings.

Among patients receiving dialysis treatment, perceived stigma represents a significant psychosocial issue that can adversely affect treatment adherence, mental well-being, social relationships, and overall quality of life (Li et al., 2024; Safi et al., 2024). Dependence on dialysis treatment, changes in body image, loss of social roles, and economic burdens may further increase perceptions of stigma among these patients. In this context, the availability of a valid and reliable measurement tool addresses an important gap in both clinical assessment and interventional research. A scale specifically designed to measure stigma among dialysis patients in Turkiye is absent, highlighting the contribution of this study to the existing literature. The Turkish version of the DRSS provides a robust instrument that can be used by nurses and other healthcare professionals to identify patients’ psychosocial needs, recognize individuals at risk, and plan interventions aimed at reducing stigma. From a clinical standpoint, evaluating stigma may assist healthcare professionals in recognizing patients susceptible to psychosocial distress. These findings may assist nurses in developing individualized care plans aimed at improving coping skills, social participation, and overall well-being (Zhuo et al., 2025).

### 4.1 Limitations

Although the Turkish version of the DRSS demonstrated adequate validity and reliability, this study was conducted in a single dialysis center, which may limit the representativeness of the sample. Besides the sample size met the minimum COnsensus-based Standards for the selection of health Measurement INstruments (COSMIN) criteria for structural validity, it did not reach the “very good” level. Therefore, the generalizability of the CFA results may be limited. Another limitation is the possibility of social desirability bias due to the face-to-face collection of data. Although this study provides evidence for content and structural validity, relationships with related psychosocial variables were not examined. Therefore, the construct validity of the scale should be interpreted within this limitation. Future studies are recommended to evaluate convergent and discriminant validity by examining associations with relevant psychosocial constructs.

In addition, the exclusion of a considerable number of participants (*n* = 30) due to severe hearing or visual impairments may have introduced selection bias and reduced the representativeness of the sample. Finally, the exceptionally high reliability coefficient observed for the Treated Differently subscale may indicate substantial item similarity or overlap. While this finding supports strong internal consistency, it should be interpreted with caution, and future research should further examine the item structure and discriminative properties of this subscale.

## 5 Conclusion

The findings of this study provide initial evidence supporting the validity and reliability of the scale in the Turkish population. However, further studies examining relationships with related psychosocial variables are needed to strengthen the construct validity of the scale. Identifying stigma-related psychosocial challenges through this instrument enables the creation of culturally appropriate, well-researched tools to identify stigma for individuals receiving hemodialysis. Additionally, the Turkish DRSS serves as a standardized assessment measure for health care providers and researchers to evaluate stigma, allowing for more precise and individualized nursing intervention strategies and the potential for developing evidence-based clinical practices focused on improving the well-being of dialysis recipients. The findings of this study provide initial evidence to support the validity and reliability of the scale within the Turkish population. However, further research examining its relationship with related psychosocial variables is required to strengthen its construct validity.

## Data Availability

The raw data supporting the conclusions of this article will be made available by the authors, without undue reservation.
